# Management of a clinical case of caries lesions by undergraduate dentistry students 

**DOI:** 10.4317/jced.61889

**Published:** 2024-12-01

**Authors:** Sebastiana Arroyo-Bote, Aina Antonelli-Sastre

**Affiliations:** 1 IDIBELL researcher. Associate Professor at the Faculty of Medicine and Health Sciences. University of Barcelona. Spain; 2 Graduate in Dentistry. Faculty of Medicine and Health Sciences. University of Barcelona. Spain

## Abstract

**Background:**

Dental caries remains the most prevalent chronic disease worldwide. Hence the importance of detecting and evaluating caries and combining this with additional diagnostic methods to ensure the best treatment. The main objective was to study what is the sensitivity and specificity for detecting initial and cavitated caries lesions by students in third, fourth and fifth year of the Degree in Dentistry at the University of Barcelona, analysing if there is any difference between the diagnoses and treatments among the different years and each student’s clinical experience. It was also determined if they apply the techniques based on the concepts of minimal intervention.

**Material and Methods:**

An observational cross-sectional study using an online survey was performed, based on a real clinical case, aimed at the students in third, fourth and fifth year of the Degree in Dentistry at the University of Barcelona.

**Results:**

Most of the respondents (72.13%) observed demineralization or cavities caused by caries in the clinical image and all in the radiographic image (100%). Meanwhile, they showed a more conservative attitude in the treatment of occlusal face, where 23.08% in 4.6 and 46.25% in 4.7 indicate filling of pits and fissures. On the other hand, on the proximal faces, they directly opted for a class II filling (95% in 4.5 distal, 94% in 4.6 mesial and 84% in 4.6 distal). Eighty-five percent of the students considered applying techniques based on the concepts of minimal intervention.

**Conclusions:**

The students use similar criteria when diagnosing and treating possible caries lesions regardless of academic year or clinical experience. The personal evaluation of the application of the concepts of minimal intervention does not correlate with the clinical attitude to those cases.

** Key words:**Dental caries, caries diagnosis, caries management, questionnaire.

## Introduction

Dental caries is a dynamic, multifactorial, non-communicable, biofilm-mediated and diet-modulated disease that results in a net loss of minerals from dental hard tissues (1). It is the most prevalent chronic disease in the world: between 60 - 90% of school-age children are affected, while in adulthood it reaches 100% (2). Its diagnosis and treatment are among the dentist’s primary tasks. According Widström *et al*. examinations, restorative treatment and anaesthesia accounted for 61.3% of all treatments, only 8.4% are Preventive measures (3).

Early detection of caries lesions is essential for non-invasive management (4), prevention and control are essential to prevent the progression to cavitation of the initial lesions, the application of fluorides stops the progression of caries in all stages of the lesion and improves the regression of the lesion (5). Therefore, a good command of the different types of diagnostic methods in daily practice as well as conducting a correct clinical evaluation of caries, classifying the severity of the lesion and evaluating its activity, are of great importance in establishing an appropriate treatment plan.

Two steps take place in the caries diagnosis process when classifying lesions: detection and evaluation. Detection refers to the objective method of deciding whether a caries lesion is present or absent, while lesion assessment characterises or monitors a lesion once detected (6,7).

The evaluation of the severity of the caries lesion is the staging of the process of net loss of minerals that progresses from small lesions to increasing degrees of dental destruction affecting the dental pulp. This assessment can be performed using a variety of classification systems and method (8).

Once the lesion is detected, it can be classified into different categories depending on the stage of progression. This method is based on visual-tactile clinical examination.

The detection of caries by visual and tactile examination is limited, particularly in cases of non-cavitated lesions, especially in proximal surfaces, which requires the use of additional diagnostic methods, among which the radiographic examination is the most frequent (9). Intraoral bitewing radiography is the most widely used test for the detection of proximal caries, being more accurate than periapical radiography and orthopantomography in the diagnosis of this type of caries (9,10). X-rays help estimate the depth of demineralisation due to caries, and a correct interpretation is essential for the accurate diagnosis of the lesion.

Although there is no single method to determine the activity of caries, Nyvad *et al*. (11) and Ekstrand *et al*. (12) laid the foundations of the current criteria to aid in assessing the activity of the lesions.

According Nyvad and Ekstrand (11,12), lesion activity is detected by evaluating the surface topography and texture of the lesions. Active enamel caries lesions are characterised as being whitish-yellowish, dull with loss of gloss, rough on tactile probe exploration, usually covered with plaque, and the gum is found with swelling and bleeding on probing close to the gingiva. Inactive lesions are bright and smooth on tactile exploration, the surface is whitish, brown or black, they are not in a plaque retention area, and the gingiva is without inflammation or bleeding on probing (11,12). On the other hand, active dentine caries lesions are soft on probing, whereas inactive ones are shiny and hard on soft probing (11,12).

Furthermore, a patient caries risk assessment can play several important roles in clinical practice, such as: helping professionals determine whether additional diagnostic procedures are required, identifying patients who need further caries control measures, evaluating the effectiveness of interventions to prevent caries, and guiding professionals in making decisions in treatment planning and in scheduling the frequency of check-ups (13). From the weighting of all the disease indicators and the risk factors with the existing protection factors, the risk of future caries appearance can be evaluated as low, moderate or high risk and, based on this, the prevention or therapeutic protocols appropriate to each case can be determined (14). However, Mejare *et al*. (15) conclude that there is a great need to standardize study design, outcome measures and reporting of data in studies on caries risk assessment because nowadays we need that an accuracy of prediction models should be validated in at least one independent population.

There are different therapeutic options depending on the degree of dental involvement as a consequence of the caries lesion, from control or non-invasive methods to invasive management. The level of intervention depends on the clinical classification of caries and the radiographic extension in addition to the assessment of the activity of the lesion. The assessment of the patient’s caries risk may also influence the therapeutic decision (4).

According to the ICCMS™ Guide for Practitioners and Educators (6) the recommended levels of clinical management for active lesions are as follows:

1. Initial lesions. Initial caries management stage: nonoperative management - control.

2. Moderate lesions. Moderate caries management stage (operative management with dental preservation).

3. Severe lesions. Stage of severe caries management (operative management with dental preservation).

For healthy tooth surfaces and inactive lesions, risk-based prevention is recommended (4).

After investigating the recommendations given by the institutions and different authors regarding the management of caries lesions, the objective of this work was to analyse University of Barcelona dentistry students in 3rd, 4th and 5th year trained in the diagnosis and treatment of lesions compatible with caries based on a clinical case.

The general objective is to study how students in third, fourth and fifth year of the Degree in Dentistry at the University of Barcelona manage the diagnosis and treatment of possible caries lesions, analysing if there are differences in relation to academic year and clinical experience.

## Material and Methods

**Figure 1:**
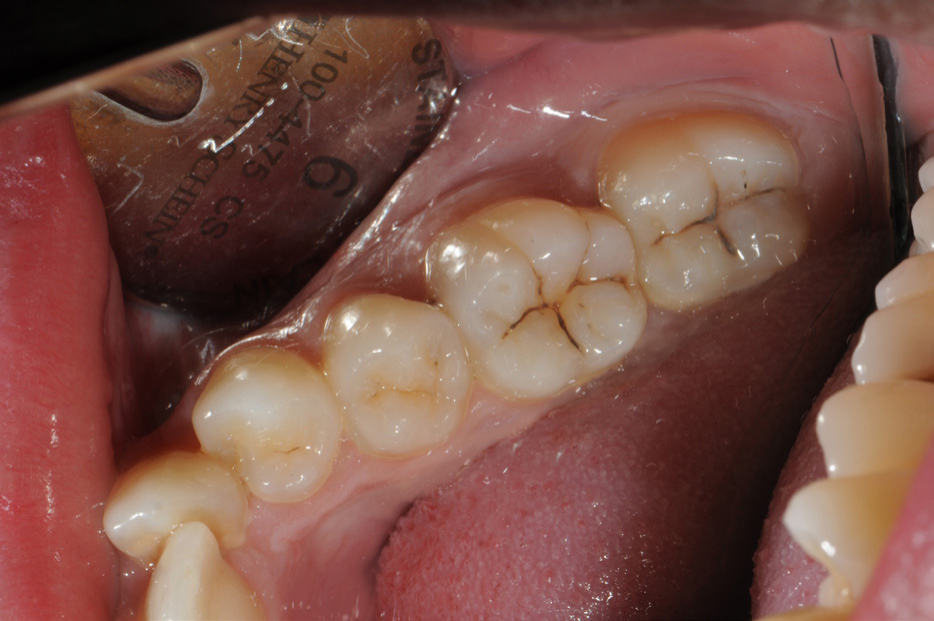
Clinical image of the fourth quadrant.

**Figure 2:**
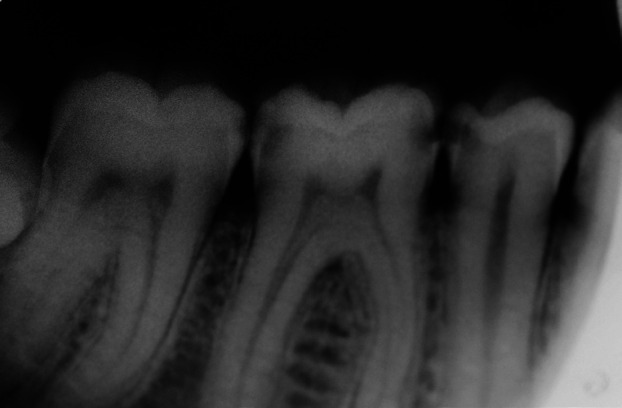
Radiographic image of the fourth quadrant.

To carry out the study, an online survey was applied, based on a real clinical case (Figs. 1,2), aimed at students in third, fourth and fifth year of the Degree in Dentistry at the University of Barcelona ( Table 1). The study protocol had been approved by the Ethics and Clinical Research Committee of the Dental Hospital of the University of Barcelona (Protocol 46/2020).

The online survey was designed using the SurveyMonkey platform and the access link to the form was sent to the students through the different WhatsApp groups of the third, fourth and fifth-year students of the Degree in Dentistry at the University of Barcelona. The form was sent between February and March 2021. There were no exclusion criteria, since the only condition was that they be enrolled in the current course.

To calculate the sample size, a proportion was estimated for a finite population of 351 students from the third, fourth and fifth years of the Degree in Dentistry at the University of Barcelona based on a 50% probability of success. For an absolute precision of ± 6% and a confidence level of 95% assuming a 10% loss of cases, a total of 169 students was required.

Before completing the survey, the participant accepted voluntary and anonymous participation in the research, authorising the statistical treatment and publication of the data ( Table 1).

Next, the student was asked about their academic year and clinical experience, asking approximately how many caries lesions of pits and fissures and of proximal faces they have treated by dental filling in the internships: 5 or fewer than 5, between 6 and 15, or more than 15.

Below are 6 multiple-choice questions about a clinical image (Fig. 1), where they were asked if they observed any caries lesions and, if so, to indicate the tooth / teeth where they think they exist and specify which treatment they would perform on each one.

Next, a radiographic image of the same case was shown (Fig. 2), and 9 multiple-choice questions were presented, asking if the participant observed any image compatible with demineralization caused by caries and, if so, to indicate the tooth / teeth where they believe they exist and specify what treatment they would perform in each case.

Finally, they were asked if they would have suspected with the initial clinical image that caries might exist on the proximal faces, and if they thought that they would apply the techniques based on the concepts of minimal intervention.

The independent variables recorded are the student’s academic year and clinical experience. On the other hand, the dependent variables are the diagnostic and treatment criteria for possible caries lesions and the application of techniques based on the concepts of minimal intervention.

The results were analysed with the “SPSS Statistics” program (IBM SPSS Statistics, version 21.0, Chicago, IL, USA). An analysis was carried out using descriptive statistics and the possible significant differences in the different variables of the study were examined. The proportions were estimated using a 95% confidence interval using the normal distribution approximation. The association between the responses and the explanatory variables was tested using the Chi-square statistical test.

The data were automatically recorded when the participant opened the link, answered the form and clicked send.

## Results

184 responses were obtained from among the third, fourth and fifth-year students in the Degree in Dentistry at the University of Barcelona, which means a participation of 52.13%.

Of the study participants, 33.52% were in third year, 31.32% in fourth and 35.16% in fifth. Regarding clinical experience, 74.18% of the students responded that they had performed filling therapy on 5 or fewer caries lesions of pits and fissures during their internship, and 64.09% had performed filling therapy on 5 or fewer on the proximal faces.

**Figure 3:**
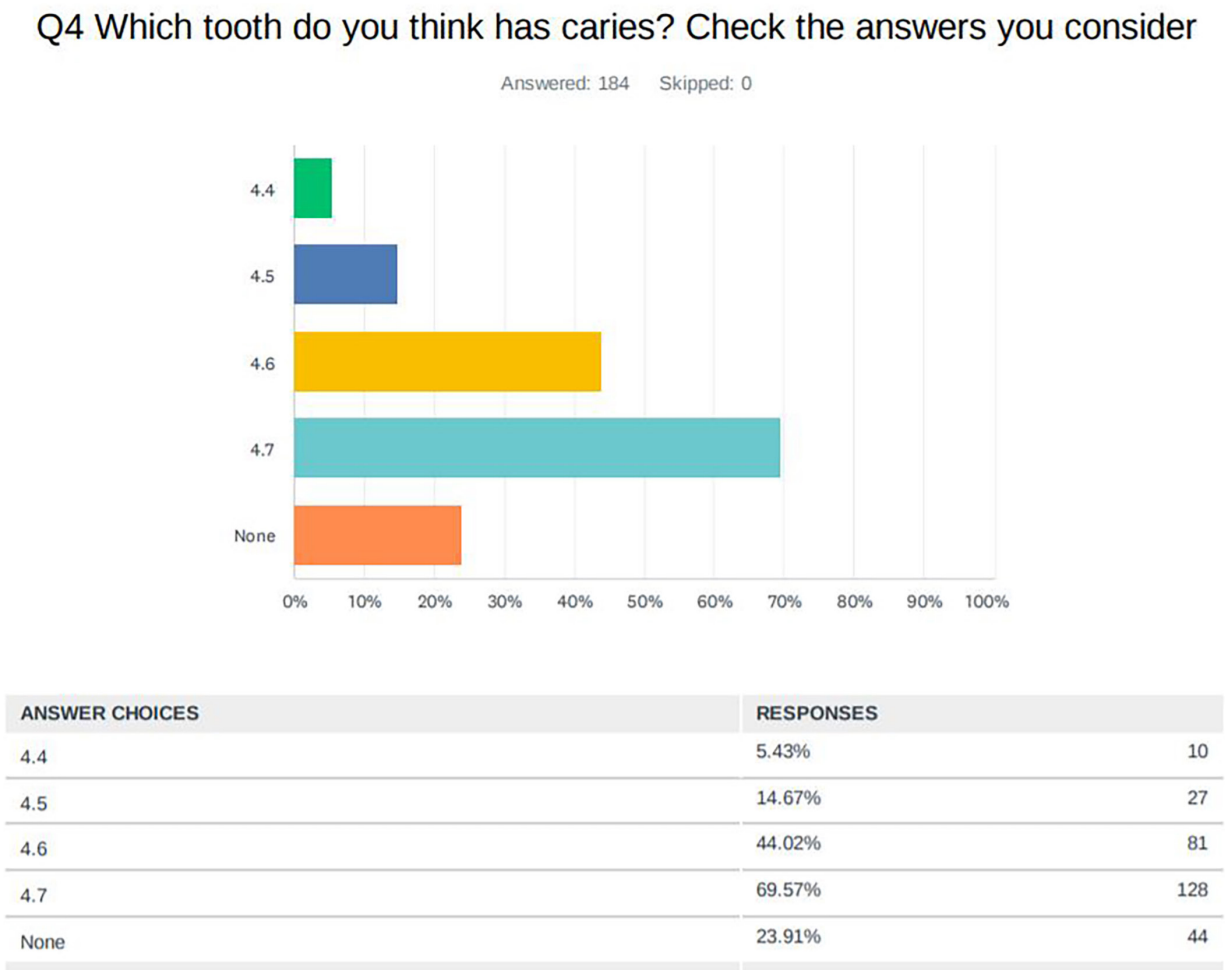
Results of the survey in relation to the diagnosis of caries in pits and fissures.

Regarding the diagnosis of possible caries lesions in pits and fissures of the clinical image of the fourth quadrant, 76.09% of the students responded that they observed some caries lesion and, in the question of which tooth / teeth they observed, 69.57% did so in 4.7, 44.02% in 4.6, 14.67% in 4.5 and 5.43% in 4.4 (Fig. 3,  Table 2)

The treatments that they consider to be the most indicated in possible caries lesions in pits and fissures vary according to the tooth, mostly indicating remineralising treatment in teeth 4.4 and 4.5 (83.61% and 82.42% respectively). 46.15% indicated dental filling treatment in 4.7 and 23.08% in 4.6, where 41.76% indicated remineralising treatment ( Table 2).

**Figure 4:**
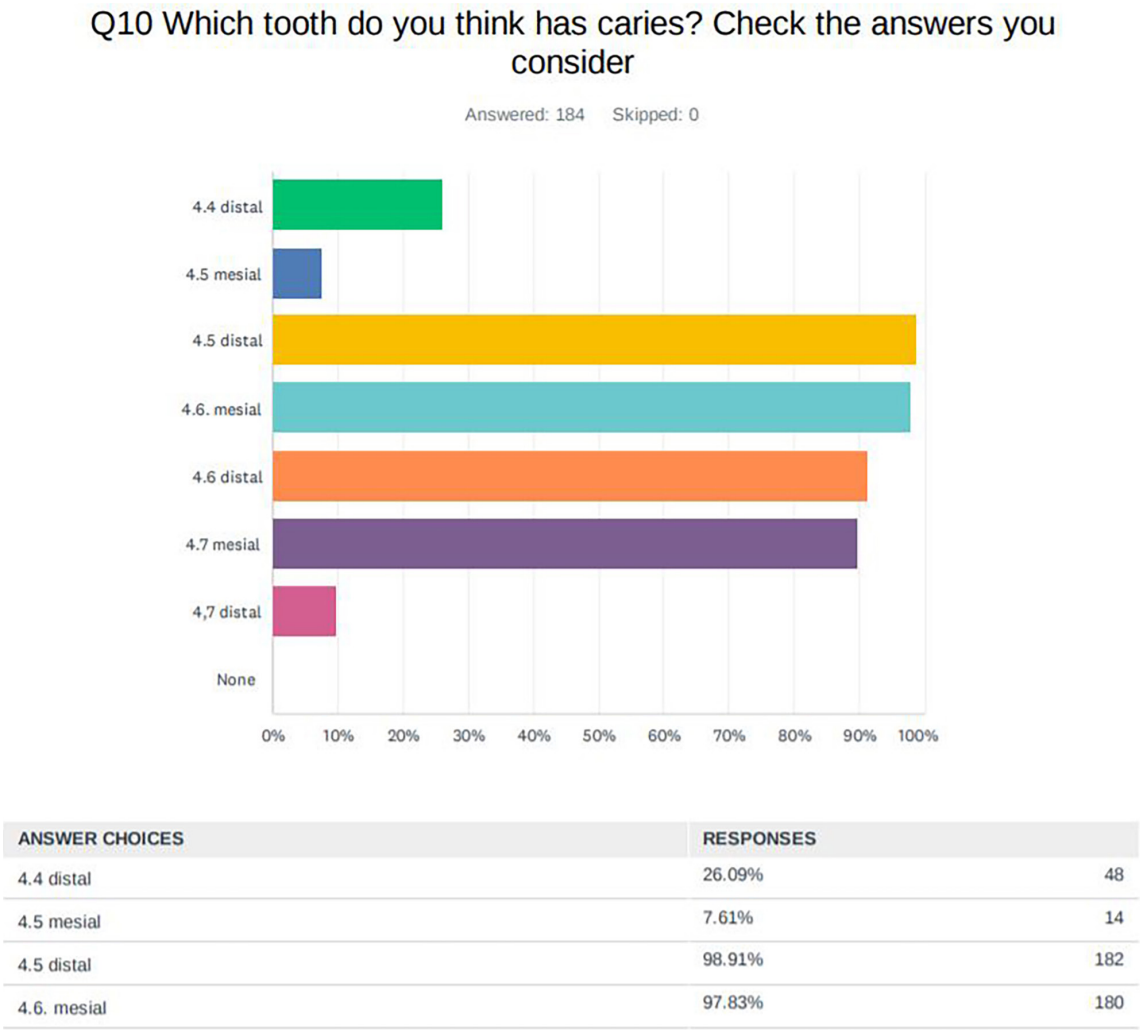
Results of the survey in relation to the diagnosis of caries in proximal faces.

In the diagnosis of caries on the proximal faces, 100% of the students answered that they observed cavities and, in the question of which tooth / teeth they observed, 98.91% located the cavities in 4.5D, followed by 97.83% in 4.6M, 91.30% in 4.6D, 89.67% in 4.7DM, 26.09% in 4.4D, 9.78% in 4.7D and 7.61% in 4.5 M (Fig. 4,  Table 2). The treatments they considered to be the most indicated varied according to the tooth and the location, mostly indicating filling or radiographic control ( Table 2).

69.95% of the students responded that they would not have suspected caries lesions on the proximal faces of the teeth with the initial clinical image.

On the other hand, 85.25% of the surveyed students responded that they considered that they apply techniques based on the concepts of minimal intervention.

Once the data analysis had been carried out, no significant difference was noted between the diagnosis of pit and fissure caries and the student’s academic year, *p-value*> 0.05, nor in relation to clinical experience. No significant differences were found in relation to the diagnosis of caries on the proximal faces and the academic year or clinical experience either.

Regarding the treatment of pits and fissures, a significant difference was seen in the treatment of tooth 4.5 and academic year: the fourth-year students would perform fewer controls (71.9%) than the third-year students (86.7%) and fifth-year students (87.5%) *P*-value <0.05 (*). A significant difference was also noted in the treatment of tooth 4.6 and academic year: students in fifth year would do less filling (9.4%) than those in third year (31.7%) and fourth year (29.8%) P-value <0.05 (*) ( Table 3). In the rest of the questions, no significant difference was observed between the treatment of caries and the student’s academic year, *p-value*> 0.05 ( Table 3).

Regarding the clinical experience and the treatment of pits and fissures, a significant difference is observed in the treatment of tooth 4.6, those who have undergone> 5 fillings plus remineralising treatment (34%) than those who have undergone <5 fillings (14, 8%) *p-value* <0.05 (*). ( Table 3). In the rest of the questions regarding the treatment of pits and fissures, no significant difference was observed between the treatment of caries and the student’s clinical experience, *p-value*> 0.05 ( Table 3).

Regarding the treatment they would use for caries lesions on the proximal faces of the teeth, a significant difference was seen in the treatment of distal tooth 4.5 and academic year: those in in third and fourth year would perform more controls (13.3% and 10.5% respectively) than those in fifth year (1.6%). Differences were also observed in the treatment of tooth 4.6 mesial and academic year, since those in third and fourth year would perform more controls (15% and 12.3% respectively) than those in fifth years (1.6%), while those in third year would perform more filling in the same tooth (100%) than those in fourth (93%) and fifth year (89.1%), *p-value* <0.05 (*). In the rest of the questions about the treatment of proximal faces, no significant difference was observed between the treatment of caries and the student’s academic year, *p-value*> 0.05 ( Table 4).

Regarding the treatment of proximal face lesions and the clinical experience, a significant difference was observed in the treatment of tooth 4.6M: the students who had performed 5 or fewer caries lesions on the proximal faces, only 1.7% would apply the treatment of resin infiltrations, while those who had carried out > 5 and, therefore had more experience, would apply it by 9.2%. Also, in tooth 4.6M, those with less experience would fill more (96.6%) than those with more experience (89.2%). We also observed a significant difference in the treatment of tooth 4.7M: those with more experience would apply more resin infiltrations (20%) than those with less (8.6%), *p-value* <0.05% (*) ( Table 4). In the rest of the questions about the treatment of proximal face lesions, no significant difference was observed between caries treatment and the student’s clinical experience, *p-value*> 0.05 ( Table 4)

No significant differences were observed between the academic year and the student’s clinical experience regarding the suspicion of caries lesions on the proximal faces of the teeth from the initial clinical image, nor in whether they considered that they apply minimal intervention techniques, *p* -value> 0.05

## Discussion

In this study, a clinical case is presented where the intraoral and radiographic image of the fourth quadrant of a patient (Figs. 1,2) is presented to diagnose possible caries lesions and, consequently, to establish the treatment if necessary.

The clinical image (Fig. 1) shows lesions on teeth 4.6 and 4.7, which, according to the definition of the ICCMSTM guide for practitioners and educators ICCMSTM categories of caries (6), denotes an ICDAS 1 0 2 case, indicating an initial stage. Assessing the appearance of the lesion, we cannot appreciate a breakage of the enamel surface (but it could be assumed microscopic cavitation) or underlying shadow in the dentine that indicates active demineralisation, so we would be facing a case that would require non-operative conservative treatment that could vary depending on the risk to the patient (13,14); however, it should not be treated with a cavity preparation. According to scientific evidence, we must accept that the evaluation of activity is imprecise and non-invasive caries control measures also have poor cost-benefit-ratio (5,11,14).

43.72% of the students considered that there were cavities in tooth 4.6 and 69.40% in tooth 4.7 (Fig. 2). However, most would perform non-operative management of the lesions: although almost 70% of the students had diagnosed caries in tooth 4.7, 54% would not perform a filling of pits and fissures. In 4.6, students in the third and fourth year would perform more operative treatments than those in fifth year, and those with more experience would apply more remineralising treatment. With this, we can say that, in this case, the students in more advanced courses and with more experience take the current criteria for the preservation of the dental structure more into consideration, lending more importance to control and prevention than to the removal of tissue.

In teeth 4.4 and 4.5 of the same clinical image, there is no evidence of visible caries, since they are healthy dental surfaces (ICDAS code 0) ( Table 1), and this was considered by most of the students surveyed (Fig. 2). The treatment would be based on risk-based caries control: clinical control of the pits and fissures would be carried out in those patients with low risk, while in those with moderate and high risk, the application of remineralising and non-invasive sealing treatments should be carried out, but the most important is encouraging to toothbrushing and avoid the intake easily fermentable saccharides. In these two cases, most of the students would opt for a clinical control of the pits and fissures: 83.61% in tooth 4.4 and 82.42% in tooth 4.5 and very few consider preventive treatments such as a remineraliser and non-invasive seals, which highlights the lack of overview of caries as a general disease, treating the teeth individually against caries.

Regarding the radiographic image (Fig. 2), radiolucencies were observed in teeth 4.5 D, 4.6 M and D and 4.7 M, and this was answered by most of the participants.

In teeth 4.5 D and 4.6 M and D, a radiolucency limited to the outer third of the dentine can be observed, which, following the ICDAS / ICCMSTM radiographic registration system (6), would be the initial stage of caries and, consequently, the recommended treatments would be the same as of the pits and fissures, emphasizing the cleaning and diet and in some cases remineralising treatment and resin infiltrations on the proximal surfaces, i.e., non-operative management. Now, in these cases where the lesion reaches the dentine, it would be appropriate to determine if there is cavitation to decide whether the treatment should be operative or not by, for example, the temporary separation of the teeth. Cavitation or any situation in the oral cavity that favours the accumulation and metabolic activity of biofilms increases the risk of the development and progression of caries (15).

Although for most dentists the criterion for restoring proximal lesions is when they reach the dentine on radiographs, most of these lesions may not be cavitated. In fact, clinical studies have shown that between 40% and 60% of the lesions found in the outer third of the dentine are not cavitated (15,16).

It has been established that the radiographic penetration depth at which the surface can be safely predicted to be cavitated and the dentine highly infected is when the radiolucency goes beyond the outer third of the dentine (5). However, according to the results of the studies by Gimenez *et al*. (17), visual inspection is the most sensitive and specific method for detecting caries at all stages in both primary and permanent teeth. Due to the superposition of healthy enamel, radiography reduces the diagnosis in the initial stages, and it is questionable to take it into consideration when deciding to carry out an invasive treatment. Since the occlusal surfaces are the most susceptible to caries, visual inspection seems to be the best method for diagnosing the disease; however, although fluorescence methods in advanced occlusal lesions may produce a better diagnosis than visual inspection, the cost of method and the scant use by dentists make this method of little importance in daily practice.

Although the students had the option of selecting multiple responses, being able to consider different treatments for these lesions, almost all (95% in tooth 4.5 D, 94% in 4.6 M and 84% in 4.6 D) opted directly for class II dental filling ( Table 3). The act of performing a restoration, particularly in proximal caries lesions, involves sacrificing considerable amounts of healthy tooth structure to gain access to the lesion area along with the removal of decayed tissues, and its durability is limited, depends on knowledge and good use of restorative materials and techniques. Therefore, microinvasive treatments must be taken into consideration (18). The fracture of the restoration or the presence of secondary caries are the main reasons for failure, which often occurs 3 years later, so monitoring the fillings is necessary for greater survival of the restoration (19).

Therefore, regardless of whether dental filling is the most indicated treatment in this case or not, since it is not possible to reliably know if these lesions are cavitated, what should be noted is that the students do not opt for the combination of this with preventive treatments to avoid the appearance of new lesions. Although preventive treatment based on scientific evidence has focused on the child as a patient, the risk of developing caries must be prevented throughout the person’s life (1).

A survey applied by Pakdaman *et al*. (20) to students of the Degree in Dentistry at the University of Sydney yielded similar results when deciding on a restorative treatment in lesions found in the external third of the dentine. In three other surveys, one carried out in Brazil and two in Scandinavia, most dentists decided to intervene operatively when the lesion reached the middle or external third of the dentine (17). In a recent survey conducted among dentists in the Balearic Islands, Spain (21), based on the same clinical case as this research, the results revealed that dentists are equally interventional in the lesions on the proximal faces, given that only 3% of them indicate resin infiltrations or remineralising treatments. A study by Baraba *et al*. (22) administered to Croatian dentists also showed similar results, as most would opt for a restoration in proximal carious lesions when these are confined to the enamel and their development can still be stopped. Raphael SL *et al*. (23), in a survey on teaching cariology in Australia and New Zealand, reported that more than 40% of respondents still teach surgical intervention for lesions limited to the enamel. However, our results show that in tooth 4.6 M, students with more clinical experience would apply more resin infiltrations and perform less filling than those with less experience.

The caries lesion of tooth 4.7 M is limited to the outer half of the enamel and, therefore, it is also an initial stage where the most indicated approach would again be remineralising treatment and resin infiltrations. 56.04% of the students would perform a class II filling ( Table 4); however, those with more experience would also apply more resin infiltrations on this occasion than those with less experience.

The 4.4 D, 4.5 M and 4.7 D teeth are healthy as there is no radiolucency in the image. It is interesting to note that 26.23% of the students responded that they observed caries in tooth 4.4 D, likely confusing it with cervical glaze or cervical wear. This phenomenon appears on radiographs as a radiolucent band around the neck of the tooth, more pronounced at the proximal edges. X-ray photons bypass or burn the thinner edge of the tooth and create this radiolucent area that mimics cervical caries. However, proximal caries lesions are more frequently found in the area of the contact point and in the free gingival margin, and do not begin below the gingival margin as occurs in cervical wear (24). Errors in the diagnosis, in this case a false positive, can lead to the unnecessary restorative treatment of healthy teeth (25). Treatment is based on risk-based prevention: in those patients with a low risk of caries, control of the proximal faces will be carried out and, in those with a moderate or high risk, a remineralising treatment will also be performed. However, 34.43% of the students would carry out an invasive treatment on tooth 4.4 D, 29.12% on 4.5 M and 12.09% on 4.7 D ( Table 4).

On the other hand, almost 70% of the students answered that they would not have suspected from the initial clinical image that caries lesions might exist on the proximal faces. This demonstrates the importance of additional diagnostic methods, such as fluorescencia and bitewing radiographs, in the diagnosis of caries, especially in proximal areas where they cannot be detected by visual inspection. Despite the most important thing being visual inspection (26), a detailed inspection of these surfaces is advisable, and visual inspection can be helped by taking photographs of proximal areas and studying them in close-up. In the clinical case of this study, photographing the proximal faces from the buccal and lingual sides could have helped to diagnose the lesions on the proximal surfaces. As no signs of demineralization were observed in the marginal ridges, however, the diagnosis of these surfaces was based on radiography which, as scientific evidence shows, can lead to diagnostic and therapeutic errors.

Intraoral bitewing radiography (27) and fluorescence study are complementary methods that aid in the diagnosis of caries, but we must bear in mind that the most sensitive and specific method in diagnosing caries is visual inspection. We should not take complementary methods as a priority when establishing invasive caries treatment, as mistakes can be made. Teeth with an intact marginal margin and healthy to the naked eye may present carious lesions to enamel and dentine (28), but it is important to avoid overtreatment that we can do due to the misinterpretation of the radiographs (29). Visual inspection has high precision values (17,26), with a tendency to greater specificity, especially in initial lesions.

Undergraduate skills in cariology in Europe are fundamentally acquired in paediatric dentistry, conservative dentistry, restorative dentistry or dental operative dentistry, beginning in 96% of cases in the third year, with preclinical exercises in 98% of schools and with clinical exercises in fourth and fifth grade in 97% of them (30). New technologies such as virtual reality or new educational adaptations aid in improving skills in cariology (31), helping broaden the systemic concept of the disease, since after analysing the results, we note that the students give less importance to preventive treatments and most focus on treating the lesions with operative management, especially in the caries lesions on proximal faces.

With this, we can say that students, in general, have not followed the holistic philosophy of caries management that defines minimally invasive dentistry, the main objective of which is the preservation of dental tissue combined with minimally invasive restorative procedures, with operative treatment being a last resort. However, 85% of the students considered that they apply these techniques based on the concepts of minimal intervention. Although the respondents may be familiar with this philosophy, effective practice is less common and, therefore, it is essential that education and practice be directed towards a caries management model that respects early detection and prevention (32). The educational scientific community is trying to ensure that caries is considered a disease, and as such it is necessary to treat the aetiology and the triggering factors and not the consequences of the disease through different consensuses in the new cariology curriculum for undergraduate students in different regions (33,34).

## Conclusions

1. Students have a similar criterion when it comes to diagnosing possible caries lesions regardless of academic year or clinical experience. Even if in some specific cases we saw a minor differences in the treatment of caries and the academic course and the clinical experience of each student, we can say that, overall, there are no differences. Therefore, they have a similar way of dealing with caries lesions.

2. Most respondents do not take preventive and microinvasive treatments, such as dental remineralisation, pit and fissure sealants and resin infiltration into consideration.

3. The education in cariology should be emphasized in the first academic years, where the student receives the first concepts in diagnosis and treatment of caries. The education of application of the concepts of minimal intervention and the clinical attitude have to go according to the scientific evidence.

4. The way in which false positives in proximal caries lesions are interpreted can lead to overtly invasive treatments.

## Figures and Tables

**Table 1:** Survey with 21 questions and answers.

Q1: Participation in this questionnaire is voluntary and anonymous. The information and conclusions drawn from the responses aim to study the preparation and behaviour of Dentistry students before clinical situations compatible with caries. The data obtained will be used for this purpose. The participant is undertaking to answer the form truthfully and authorizes the statistical treatment and analysis of the results for teaching.	Yes									No					
Q2: What academic year are you taking?	Third course				fourth course							fifth course			
Q3: Do you see any caries lesions in the following clinical image of the fourth quadrant? (Figure 1)	Yes				No							Do not know, no answer			
Q4: Which tooth do you think has cavities? Check the answers that consider	4.4	4.5						4.6				4.7		None	
Q5: What treatment would you carry out in 44? Check the answers that consider	Clinical control	Remineralizing treatment						Non-invasive sealing				Invasive sealing		Class I Filling	
Q6: What treatment would you carry out in 45? Check the answers that consider	Clinical control	Remineralizing treatment						Non-invasive sealing				Invasive sealing		Class I Filling	
Q7: What treatment would you carry out in 46? Check the answers that consider	Clinical control	Remineralizing treatment						Non-invasive sealing				Invasive sealing		Class I Filling	
Q8: What treatment would you carry out in 47? Check the answers that consider	Clinical control	Remineralizing treatment						Non-invasive sealing				Invasive sealing		Class I Filling	
Q9: Can you identify any images on the X-ray compatible with a lesion of caries? (Figure 2)	Yes			No								Do not know, no answer			
Q10: Which tooth do you think has cavities? Check the answers thatconsider	4.4D	4.5M		4.5D			4.6M		4.6D		4.7M		4.7D		None
Q11: What treatment would you carry out in the 44D? Check the answers you consider	Radiographic control		Remineralizing treatment						Resin infiltrations					Class II Filling	
Q12: What treatment would you carry out in the 45M? Check the answers you consider	Radiographic control		Remineralizing treatment						Resin infiltrations					Class II Filling	
Q13: What treatment would you carry out in the 45D? Check the answers you consider	Radiographic control		Remineralizing treatment						Resin infiltrations					Class II Filling	
Q14: What treatment would you carry out in the 46M? Check the answers you consider	Radiographic control		Remineralizing treatment						Resin infiltrations					Class II Filling	
Q15: What treatment would you carry out in the 46D? Check the answers you consider	Radiographic control		Remineralizing treatment						Resin infiltrations					Class II Filling	
Q16: What treatment would you carry out in the 47M? Check the answers you consider	Radiographic control		Remineralizing treatment						Resin infiltrations					Class II Filling	
Q17: What treatment would you carry out in the 47D? Check the answers you consider	Radiographic control		Remineralizing treatment						Resin infiltrations					Class II Filling	
Q18: Would you have suspected from the initial clinical image that there might be caries lesions on the proximal sides of the teeth?	Yes					No					Do not know, no answer				
Q19: How many pit and fissure caries lesions have you performed in	<5					6-15					>15				
Q20: How many caries lesions on the proximal faces have you made in practices?	<5					6-15					>15				
Q21: Do you think that you apply techniques based on the concepts of minimum intervention, that is, prioritizes the preservation of dental tissue, including early detection of caries and non-invasive treatment?	Yes					No					Do not know, no answer				

**Table 2:** Survey results in the Diagnosis and Treatment of occlusal and proximal lesions.

Tooth Clinic Image (Figure 1)	Diagnosis of caries	Clinical control	Remineralizing treatment	Non-invasive sealing	Invasive sealing	Class I Filling
4.4	5.43%	83.61%	14.21%	17.49%	1.64%	1,09%
4.5	14.67%	82.42%	11.54%	17.03%	1.65%	4,40%
4.6	43.02%	41.76%	19.78%	23.08%	15.93%	23,08%
4.7	69.57%	29.12%	14.29%	13.19%	18.13%	46,15%
None	23.91%					
Tooth Radiographic Image (Figure 2)	Diagnosis of caries	Radiographic control	Remineralizing treatment	Resin infiltrations		Class II Filling
4.4 Distal	26.09%	62.84%	8.20%	3.28%		34.43%
4.5 Mesial	7.61%	68.13%	9.34%	1.65%		29.12%
4.5 Distal	98.91%	8.24%	2.75%	4.40%		95.05%
4.6 Mesial	97.83%	9.34%	2.75%	4.40%		93.96%
4.6 Distal	91.30%	17.58%	8.24%	6.59%		84.07%
4.7 Mesial	89.67%	26.37%	25.27%	12.64%		56.04%
4.7 Distal	9.78%	86.26%	4.95%	2.75%		12.09%
None	0.00%					

**Table 3:** Chi-square tests: treatment of caries in pits and fissures and academic year.

Variable		Chi-square value	Degrees of freedom	P-value	Chi-square value	Degrees of freedom	P-value
4.4	Control	2.688	2	0.261	0.116	1	0.733
	Remineralizing treatment	0.931	2	0.628	0.072	1	0.789
	Not invasive sealing of pits and fissures	4.796	2	0.091	0.014	1	0.907
	Invasive sealing of pits and fissures	0.007	2	0.997	0.090	1	0.764
	Filling	1.013	2	0.603	0.704	1	0.401
4.5	Control	6.187	2	0.045*	3.599	1	0.058
	Remineralizing treatment	0.510	2	0.775	0.510	1	0.199
	Not invasive sealing of pits and fissures	4.354	2	0.113	0.816	1	0.366
	Invasive sealing of pits and fissures	No statistics calculated because 100% of students would not perform this treatment.			No statistics calculated because 100% of students would not perform this treatment.		
	Filling	3.886	2	0.143	0.776	1	0.379
4.6	Control	2.635	2	0.268	0.814	1	0.367
	Remineralizing treatment	3.860	2	0.145	8.123	1	0.004*
	Not invasive sealing of pits and fissures	1.198	2	0.549	0.116	1	0.734
	Invasive sealing of pits and fissures	0.423	2	0.809	0.489	1	0.484
	Filling	10.682	2	0.005*	2.390	1	0.122
4.7	Control	1.704	2	0.427	0.066	1	0.798
	Remineralizing treatment	0.775	2	0.679	0.387	1	0.534
	Not invasive sealing of pits and fissures	0.564	2	0.754	0.010	1	0.921
	Invasive sealing of pits and fissures	1.567	2	0.457	0.422	1	0.516
	Filling	0.377	2	0.828	1.574	1	0.210

(2) and Chi-square tests: treatment of caries in pits and fissures and clinical experience (1) * significant difference *p*<0.05.

**Table 4:** Chi-square tests: caries treatment on proximal faces and academic course.

Variable		Chi-square value	Degrees of freedom	P-value	Chi-square value	Degrees of freedom	P-value
4.4D	Radiographic control	2.015	2	0.365	0.009	1	0.923
	Remineralizing treatment	0.221	2	0.896	0.119	1	0.730
	Resin infiltrations	0.014	2	0.993	0.999	1	0.318
	Class II Filling	1.556	2	0.459	0.907	1	0.341
4.5M	Radiographic control	0.169	2	0.919	2.223	1	0.136
	Remineralizing treatment	0.834	2	0.659	0.344	1	0.557
	Resin infiltrations	5.577	2	0.062	0.009	1	0.925
	Class II Filling	2.389	2	0.303	2.561	1	0.110
4.5D	Radiographic control	6.194	2	0.045*	0.000	1	0.987
	Remineralizing treatment	0.534	2	0.766	0.037	1	0.847
	Resin infiltrations	0.274	2	0.872	0.722	1	0.396
	Class II Filling	2.113	2	0.348	1.588	1	0.208
4.6M	Radiographic control	7.386	2	0.025*	0.166	1	0.684
	Remineralizing treatment	0.320	2	0.852	1.296	1	0.255
	Resin infiltrations	2.932	2	0.231	5.557	1	0.018*
	Class II Filling	6.619	2	0.037*	3.912	1	0.048*
4.6D	Radiographic control	8.984	2	0.011	0.217	1	0.641
	Remineralizing treatment	2.068	2	0.356	0.119	1	0.730
	Resin infiltrations	1.676	2	0.433	2.807	1	0.094
	Class II Filling	1.925	2	0.384	2.294	1	0.130
4.7M	Radiographic control	1.132	2	0.568	0.157	1	0.692
	Remineralizing treatment	4.280	2	0.118	0.278	1	0.498
	Resin infiltrations	2.209	2	0.331	4.863	1	0.027*
	Class II Filling	12.353	2	0.002*	3.827	1	0.050
4.7D	Radiographic control	2.060	2	0.357	1.842	1	0.175
	Remineralizing treatment	0.480	2	0.787	0.027	1	0.869
	Resin infiltrations	0.418	2	0.812	0.466	1	0.452
	Class II Filling	1.228	2	0.541	0.991	1	0.320

(2) * significant difference *p*<0.05. Chi-square tests: caries treatment on proximal faces and clinical experience (1) * significant difference *p*<0.05.

## Data Availability

The datasets used and/or analyzed during the current study are available from the corresponding author.
